# Hemoglobin Genotypes Modulate Inflammatory Response to *Plasmodium* Infection

**DOI:** 10.3389/fimmu.2020.593546

**Published:** 2020-12-23

**Authors:** Keri Oxendine Harp, Felix Botchway, Yvonne Dei-Adomakoh, Michael D. Wilson, Joshua L. Hood, Andrew A. Adjei, Jonathan K. Stiles, Adel Driss

**Affiliations:** ^1^Department of Physiology, Morehouse School of Medicine, Atlanta, GA, United States; ^2^Department of Pathology, Korle-Bu Teaching Hospital, University of Ghana Medical School, Accra, Ghana; ^3^Department of Haematology, University of Ghana Medical School, Accra, Ghana; ^4^Department of Parasitology, Noguchi Memorial Institute for Medical Research, University of Ghana, Accra, Ghana; ^5^Department of Pharmacology and Toxicology & the James Graham Brown Cancer Center & the Hepatobiology and Toxicology COBRE, University of Louisville, School of Medicine, Louisville, KY, United States; ^6^Department of Microbiology, Biochemistry and Immunology, Morehouse School of Medicine, Atlanta, GA, United States

**Keywords:** sickle cell disease, cytokines, chemokines, biomarkers, sickle cell trait, malaria severity, Hemoglobin C, cerebral malaria

## Abstract

In 2018, 228 million cases and 405,000 malaria-associated deaths were reported worldwide with a majority being in Africa. A wide range of factors, including parasitemia, host immunity, inflammatory responses to infection, and host hemoglobin genotype, mediate the severity of malaria. Among the hemoglobinopathies, hemoglobin S (HbS) is caused by a single amino acid substitution of Glutamic Acid replaced by Valine at the sixth position of the beta-globin chain (E6V). Hemoglobin C (HbC) on the other hand, involves a single amino acid substitution of Glutamic Acid by a Lysine (E6K), which has received the most attention. These substitutions alter the stability of Hb leading to wide-ranging hematological disorders. The homozygous state of hemoglobin S (HbSS) results in sickle cell anemia (SCA) whereas the heterozygous state (HbAS) results in sickle cell trait (SCT). Both mutations are reported to mediate the reduction in the severity and fatality of *Plasmodium falciparum* malaria. The mechanism underlying this protection is poorly understood. Since both malaria and sickle cell disease (SCD) are associated with the destruction of erythrocytes and widespread systemic inflammation, identifying which inflammatory factor(s) mediate susceptibility of individuals with different hemoglobin genotypes to *Plasmodium* infection could result in the discovery of new predictive markers and interventions against malaria or SCD severity. We hypothesized that hemoglobin genotypes modulate the inflammatory response to *Plasmodium* infection. We conducted a cross-sectional study in Ghana, West Africa, between 2014 and 2019 to ascertain the relationships between blood inflammatory cytokines, *Plasmodium* infection, and hemoglobin genotype. A total of 923 volunteers were enrolled in the study. A total of 74, age and sex-matched subjects were identified with various genotypes including HbAS, HbAC, HbSS, HbSC, HbCC, or HbAA. Complete blood counts and serum inflammatory cytokine expression levels were assessed. The results indicate that differential expression of CXCL10, TNF-α, CCL2, IL-8, and IL-6 were tightly linked to hemoglobin genotype and severity of *Plasmodium* infection and that these cytokine levels may be predictive for susceptibility to severe malaria or SCD severity.

## Introduction

An estimated 228 million cases and 405,000 deaths associated with malaria worldwide were reported in 2018 with 93% of the cases occurring in Africa (1). Sixty-seven percent (67%) of the deaths occurred among children under the age of 5 years (1). Malaria is caused by *Plasmodium* parasites that are carried by female *Anopheles* mosquitos (1). However, malaria severity can vary from asymptomatic to uncomplicated (mild), to the severe disease associated with increasing mortality (2). The variation in severity has been attributed to a wide range of factors including parasitemia, host immunity, inflammatory responses to infection, and host hemoglobin (Hb) genotype (2–4). Among the abnormal hemoglobin’s (hemoglobinopathies), hemoglobin S (HbS) is caused by a single amino acid substitution of Glutamic Acid replaced by Valine at the sixth position of the beta-globin chain (E6V) (5). This substitution is a result of a single nucleotide substitution, GAG→GTG in codon 6 of the beta-globin gene on chromosome 11p15.5 (6). Alternatively, hemoglobin C (HbC), is associated with a mutation in the same codon GAG→AAG, which causes a single amino acid substitution of Glutamic Acid by a Lysine (E6K) (5, 6). These mutations alter the stability of Hb resulting in a variety of clinical symptoms. The homozygous hemoglobin S and C (HbSS & HbSC) genotypes result in sickle cell anemia (SCA) whereas heterozygous hemoglobin S and C genotypes (HbAS and HbAC) result in sickle cell trait (SCT). SCT reportedly mediates protection against severe and fatal forms of *Plasmodium falciparum* malaria (2, 7). Individuals with sickle cell trait (SCT), have the lowest mortality rates for malaria (2) and rarely manifest complications associated with the HbS or HbC allele (7). The molecular mechanisms mediating the resistance of SCT individuals to malaria are unclear. Sickle cell anemia patients suffer from a wide range of clinical complications including stroke, chronic infections, and acute splenic sequestration due to structural changes in RBCs under hypoxic conditions (8). Individuals with homozygous hemoglobin C genotype (HbCC) can be asymptomatic (9). The mechanisms underlying this protection are poorly understood. Malaria and sickle cell disease (SCD) are associated with the destruction of erythrocytes and widespread systemic inflammation. Identifying the inflammatory factor(s) mediating susceptibility of individuals with different hemoglobin genotypes to *Plasmodium* infection could lead to the discovery of new predictive markers and interventions against malaria or SCD severity (1, 2, 8, 10, 11).

Human immune responses to infections result in major alterations in host inflammatory cytokine profiles which play a key role in pathogenesis, destruction of invading pathogens, and the development of long- and short-term immunity (12, 13). Pro-inflammatory or anti-inflammatory cytokines are signaling molecules secreted from helper T (T_h_), natural killer, monocytes, macrophages, and other cell types to promote inflammation (14). They include interleukin-1 (IL-1), IL-12, and IL-18, tumor necrosis factor alpha (TNF-α), interferon gamma (IFNγ), and granulocyte-macrophage colony stimulating factor (GM-CSF) which contribute to innate immune responses (14). Increased production of pro-inflammatory cytokines in response to different diseases such as atherosclerosis, cancer, obesity, and SCD alter the balance between proinflammatory and anti-inflammatory cytokines necessary to maintain health physiological states (10, 13, 15, 16). Chemotactic cytokines [inflammatory chemokines (14, 17)] also participate in disease pathogenesis (i.e. pro-inflammatory stimuli, such as IL-1, TNF-α, LPS, viruses or parasites such as malaria) by actively attracting immune cells to sites of inflammation (12, 13, 18). Pro-inflammatory chemokines (CXCL10, CCL2, and CCL3) and cytokines (TNF-α, IL-8, and IL-6) were reported to mediate severe malaria and SCD separately but have not been examined in SCD individuals infected with malaria.

We hypothesized that changes in pro-inflammatory cytokines/chemokine expression normally associated with different sickle cell genotypes would correlate susceptibility or resistance to severe malaria. We conducted a cross-sectional study to determine whether levels of plasma pro-inflammatory cytokine/chemokine in individuals with different hemoglobin genotypes infected with or without *P. falciparum* will correlate with parasite growth rates. Blood samples were obtained from volunteers in Accra, Ghana, following IRB approval and consent of subjects between 2014 and 2018. The most common hemoglobinopathies in West Africa are associated with the HbC and HbS genotypes (5). Approximately 30% of Ghanaians have SCT and about 1.9% of births per year have SCD (19, 20). Ghana accounts for 4% of the global burden of malaria and 7% of the West African malaria burden (1).

We evaluated the expression of CXCL10, TNF-α, CCL2, CCL3, IL-8, and IL-6 among 74 volunteers, from a pool of 923, with one (HbAS and HbAC), two (HbSS, HbSC, HbCC) and no (HbAA) copies of the Hb allele variant S and/or C with (+) and without (-) malaria. This exploratory study provides a new opportunity to assess how alterations in cytokine/chemokine profiles by certain comorbidities may contribute to susceptibility to malaria. As well as the potential discovery of new biomarkers for assessing malaria or SCD severity and new interventions.

## Methods

### Study Population

Volunteers from the Greater Accra region in Ghana, West Africa were enrolled from multiple hospitals and polyclinics: Korle-Bu Teaching Hospital, Ghana Institute of Clinical Genetics, Korle-Bu Polyclinics, and district hospitals namely, Princess Marie Louise Children’s Hospital, Mamprobi Polyclinic, Ussher Polyclinic, and La General Hospital. A total of 923 samples were collected between February and November 2014 and June 2017 to July 2019 as part of an ongoing NIH-funded SCD and malaria study at Morehouse School of Medicine, Atlanta GA, USA, and in collaboration with the University of Ghana.

This study was approved by the ethics review boards of Morehouse School of Medicine, College of Health Sciences, University of Ghana, and the Noguchi Memorial Institute for Medical Research at University of Ghana. The study was approved at all collaborating institutions before the commencement of the study. Participants over the age of 18 years gave informed consent.

Individuals with high Fetal Hb (HbF), pregnant women, HIV+, and individuals with partial or missing information in their files relating to Complete Blood Counts (CBC) were excluded from the study. Samples were numerically coded. A total of 74, age and sex-matched, subjects were randomly selected from the pool of 923 individuals representing all hemoglobin genotypes either positive or negative for malaria. Eight (8) subjects each were selected from four (4) genotype groups (HbAA-, HbAA+, HbSS+, HbCC-) and six (6) subjects each from seven (7) genotype groups (HbAS-, HbAS+, HbSS-, HbAC-, HbAC+, HbSC-, HbSC+). The HbCC+ genotype was extremely rare and was not included in the pool. The groups were then age and gender-matched, with the average age being close to 30 years for all groups.

### Laboratory Evaluation of Blood Samples

All blood samples were collected in BD Vacutainer® CPT™ tubes (BD Bioscience, San Jose, CA). Mononuclear white blood cells (WBCs), plasma, and red blood cells (RBCs) were separated from the CPT tubes within an 8 h window, according to the manufacturer’s instructions. Hemoglobin status was determined using cellulose acetate membrane electrophoresis at the Department of Hematology at the Korle-Bu Teaching Hospital (21). Hematological characteristics were assessed by CBC at the hospital’s clinical pathology and hematology laboratories. Malaria status was determined using *Plasmodium falciparum* Rapid Diagnostic Test kits and thick smear microscopy. HIV status was also assessed using HIV Rapid Diagnostic Tests.

### Multiplexed Immunoassay

Plasma cytokine concentrations were determined using Bio-Plex Pro Human Cytokine Group I Panel (Bio-Rad Cat#:M500KCAF0Y) which targeted 27 cytokines: Interleukin (IL)-1β, IL-1ra, IL-2, IL-4, Il-5, IL-6, IL-7, Il-8, IL-9, IL-10, IL-12, IL-13, IL-15, IL-17A, CXCL10, CCL2, MIP-1α, MIP-1β, PDGF-BB, RANTES, TNF-α, VEGF, FGF basic, Eotaxin, G-CSF, GM-CSF, and IFN-γ. The plates were analyzed by a multiplexed microsphere cytokine immunoassay procedure (Luminex LX100) at Emory University’s Peds/Winship Flow Cytometry Core.

### Statistical Analysis

All statistical analyses were done in GraphPad PRISM version 7.04 for Windows (GraphPad Software, La Jolla California) unless otherwise stated. A sample size calculation using preliminary results determined a minimum of 3 samples per group were needed using a 95% confidence interval and power of 80 by using preliminary multiplexed microsphere cytokine immunoassay data. Study samples and hematologic profiles were stratified by Hb subtypes and normalized by the D’Agostino & Pearson normality test. Significant differences within and between groups were assessed for cytokine levels and CBC levels using a one-way ordinary ANOVA and Tukey’s multiple comparison tests. Cytokine concentrations that remained unchanged and those that fell out of linear range were excluded. An unpaired two-tailed t-test or two-tailed Mann-Whitney test was used to assess significant differences between two groups for parametric or nonparametric data, respectively (**Supplement Figure 1**, **Supplement Table 3**). Assessment of ratios of circulating cytokine concentrations in different hemoglobin variants with and without malaria enables the detection of significant perturbations in host cytokine profiles attributed to their hemoglobin status. Ratios of cytokine concentrations can also be used to develop algorithms for predicting susceptibility or resistance to malaria. Receiver Operating Characteristic (ROC) curves were used to assess the ability of a test to discriminate between groups. The area under ROC curves (AUC) evaluated the specificity and sensitivity of a test to differentiate between the groups (22). Statistical significance was pegged at p <0.05 for all tests. Principle Component Analysis using Past 3.x (23) was applied to the cytokine targets and their respective concentrations (CXCL10, CCL2, TNF-α, CCL3, IL-8, IL-6) and CBC [WBC, RBC, Hb, and platelet (PLT)]. A correlation matrix analysis was performed to assess between-group variable. For the PCA analysis, missing data were replaced with column average using mean value imputation.

## Results

### Complete Blood Counts Are Altered in Individuals With Different Hemoglobin Genotypes Based on Malaria Status

Age, WBC, RBC, Hb, and PLT levels were assessed and compared among all hemoglobin genotypes with and without malaria (**Table 1** & **Supplement Table 1**). There were no age effects between groups. There were significant differences in WBC counts especially for the HbSS+ group vs the following groups: HbAA- (P < 0.0001), HbAA+ (P < 0.0001), HbAC- (P < 0.0001), HbAC+ (P = 0.0001), HbAS- (P < 0.0001), HbAS+ (P < 0.0001), HbCC- (P < 0.0001), and HbSC- (P = 0.0008). RBC counts were significantly different between HbSS- vs the following groups: HbAA- (P = 0.01), HbAC- (P = 0.04), HbAC+ (P = 0.02), HbAS- (P = 0.001), and HbCC- (P = 0.04); as well as HbSS+ vs the groups: HbAA- (P < 0.0001), HbAA+ (P = 0.008), HbAC- (P = 0.0002), HbAC+ (P < 0.0001), HbAS- (P < 0.0001), HbAS+ (P = 0.001), HbSC- (P = 0.005), and HbCC- (P = 0.0001). Similarly, and as expected in hemoglobinopathies and anemic malaria, significant differences were observed for Hb and PLT level between several Hb genotype groups with and without malaria for example for Hb there was significant differences between HbAA- vs HbAA+ (P = 0.03) and HbSS- vs HbAA- (P < 0.0001). All the other significant group comparisons are shown in **Supplement Table 1**.

**Table 1:** Participant clinical characteristics.

Mean±SD	HbAA- n = 8	HbAA+ n = 8	HbAS- n = 6	HbAS+ n = 6	HbSS-n = 6	HbSS+ n = 8	HbAC- n = 6	HbAC+ n = 6	HbSC-n = 6	HbSC+ n = 6	HbCC- n = 8
Sex (males)	4	4	3	3	2	4	3	3	3	3	4
Age (years)	30 ± 6.3	30.1 ± 8.7	29.8 ± 5.7	33 ± 19.5	27.8 ± 7.4	24.4 ± 3.8	31.8 ± 7.4	26.8 ± 12.5	30.2 ± 3	34.7 ± 16	32.8 ± 9.2
WBC (x10^3^/mm^3^)	5.3 ± 1.1	5.1 ± 0.8	5.6 ± 1.4	5.9 ± 2.3	10.9 ± 1.5	18.3 ± 9.7	5.1 ± 0.7	6.5 ± 1.3	7.7 ± 1.3	12 ± 8.4	7.3 ± 1.5
RBC (x10^6^/µl)	4.9 ± 0.6	4.1 ± 0.8	5.3 ± 1.2	4.4 ± 0.8	3.1 ± 0.5	2.4 ± 1.1	4.8 ± 0.9	4.8 ± 1	4.3 ± 0.6	3.7 ± 0.9	4.6 ± 0.6
Hb (g/dl)	14.3 ± 2.5	10.5 ± 2.3	15.2 ± 3.4	12.2 ± 1.9	7.9 ± 0.79	6.05 ± 1.5	13 ± 3.1	12 ± 1.4	10.6 ± 1.1	9.5 ± 2.4	11.6 ± 1.5
PLT (x10^3^/µl)	293.6 ± 50.42	215 ± 130.5	278.5 ± 111.7	214.5 ± 79.4	436.5 ± 89.79	415.9 ± 122.2	277.5 ± 117.5	143.5 ± 71.7	323 ± 143.3	239.7 ± 166.8	223.1 ± 61.31

Mean and standard deviation for clinical characteristics for all genotypes with (+) and without (-) malaria.

### Cytokine Levels in Individuals With Different Hemoglobin Genotypes Are Altered Based on Their Malaria Status

**Figure 1:**
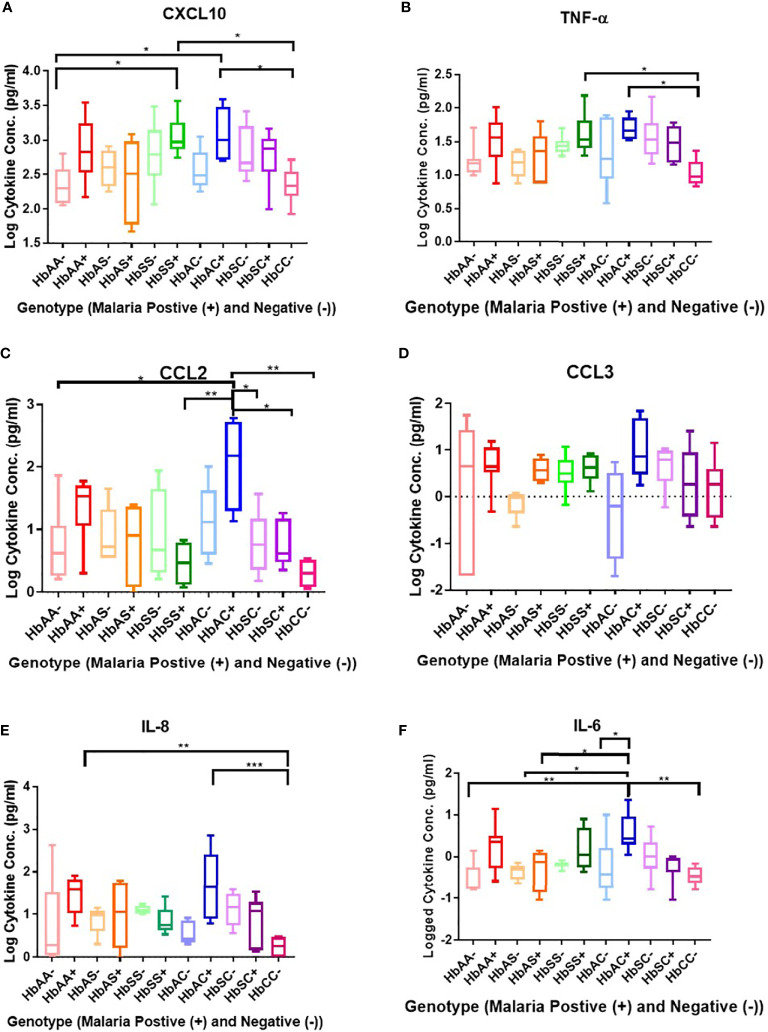
Comparisons between Hb genotypes and inflammatory cytokines with and without *Plasmodium* infection. A one-way ANOVA and Tukey’s multiple comparison test was used to assess between all the genotypes with (+) and without (-) malaria. Graphs are box and whisper plots showing minimum and maximum values. **(A)** Assessment of CXCL10 in different Hb genotypes that were infected or uninfected with malaria: HbAA- vs HbSS+ (P = 0.01), HbAA- vs HbAC+ (P = 0.02), HbSS+ vs HbCC- (P = 0.01), and HbAC+ vs HbCC- (P = 0.02). **(B)** Assessment of CCL2 in different Hb genotypes that were infected or uninfected with malaria: HbAA- vs HbAC+ (P = 0.02), HbAC+ vs HbSS+ (P = 0.007), HbAC+ vs HbSC- (P = 0.04), HbAC+ vs HbSC+ (P = 0.04), and HbAC+ vs HbCC- (P = 0.002). **(C)** Assessment of TNF-α in different Hb genotypes that were infected or uninfected with malaria: HbCC- vs HbSS+ (P = 0.04) and HbCC- vs HbAC+ (P = 0.02). **(D)** Assessment of CCL3 in different Hb genotypes that were infected or uninfected with malaria: no significant differences observed. **(E)** Assessment of IL-8 in different Hb genotypes that were infected or uninfected with malaria: HbAA+ vs HbCC- (P = 0.005) and HbAC+ vs HbCC- (P = 0.0009). **(F)** Assessment of IL-6 in different Hb genotypes that were infected or uninfected with malaria: HbAA- vs HbAC+ (P = 0.004), HbAS- vs HbAC+ (P = 0.02), HbAS+ vs HbAC+ (P = 0.03), HbAC- vs HbAC+ (P = 0.044), and HbAC+ vs HbCC- (P = 0.002). *: 0.049-0.01, **: 0.009-0.001, ***: ≤0.0009.

Plasma cytokine concentrations in individuals with different hemoglobin genotypes that were infected (+) or uninfected (-) with malaria parasites were analyzed between and among groups (**Figure 1**; **Supplement Table 2**). CXCL10 (C-X-C Motif Chemokine Ligand 10 or IP10) concentration was significantly different between hemoglobin genotypes; HbAA- vs HbSS+ (P = 0.01), HbAA- vs HbAC+ (P = 0.02), HbSS+ vs HbCC- (P = 0.01), and HbAC+ vs HbCC- (P = 0.02) (**Figure 1A**). TNF-α (Tumor necrosis factor alpha) concentration was significantly altered among hemoglobin genotypes; HbCC- vs HbSS+ (P = 0.04) and HbCC- vs HbAC+ (P = 0.02) (**Figure 1B**). CCL2 (C-C Motif Chemokine Ligand 2) concentration was altered between hemoglobin genotypes; HbAA- vs HbAC+ (P = 0.02), HbAC+ vs HbSS+ (P = 0.007), HbAC+ vs HbSC- (P = 0.04), HbAC+ vs HbSC+ (P = 0.04), and HbAC+ vs HbCC- (P = 0.002) (**Figure 1C**). IL-8 (Interleukin 8) concentration was also altered between HbAA+ vs HbCC- (P = 0.005) and HbAC+ vs HbCC- (P = 0.0009) (**Figure 1E**). We determined that there were no significant differences between CCL3 (C-C Motif Chemokine Ligand 3) concentrations in genotype groups with or without malaria (**Figure 1D**). IL-6 concentration was altered between HbAA- vs HbAC+ (P = 0.004), HbAS- vs HbAC+ (P = 0.02), HbAS+ vs HbAC+ (P = 0.03), HbAC- vs HbAC+ (P = 0.044), and HbAC+ vs HbCC- (P = 0.002) (**Figure 1F**).

There were no significant differences between males and females for all cytokines (figure not shown). CXCL10 concentration was significantly different between HbAA- vs HbSS- (P = 0.04), HbSC- (P = 0.02), HbAA+ (P = 0.01) (**Supplement Figure 1A**), HbSS+ (P = 0.0001), and between HbAC- vs HbAC+ (P = 0.03) (**Supplement Figure 1B**, **Supplement Table 3**). Similarly, there was a significant difference for TNF-α levels between HbAA- vs HbSS- (P = 0.03) (**Supplement Figure 1D**) and of CCL2 levels between HbAA- vs HbSS+ (P = 0.04) (**Supplement Figure 1C**). Similarly, significant differences were observed for CCL3 comparing HbAC- vs HbAC+ (P = 0.03) and HbAS- vs HbAS+ (P = 0.008) (**Supplement Figure 1E**) and for IL-8 comparing HbAC- vs HbAC+ (P = 0.01) and HbAA+ vs HbSS+ (P = 0.007) (**Supplement Figure 1F**). Finally, there were a significant difference between HbAA+ vs HbAA- (P = 0.01) and HbAC- vs HbAC+ (P = 0.03) for IL-6 (**Supplement Figure 1G**).

### Receiver Operating Characteristic Curve Analysis of Circulating Concentrations of CXCL10, TNF-α, CCL2, CCL3, and IL-8 in Individuals With Different Hemoglobin Genotypes

**Figure 2:**
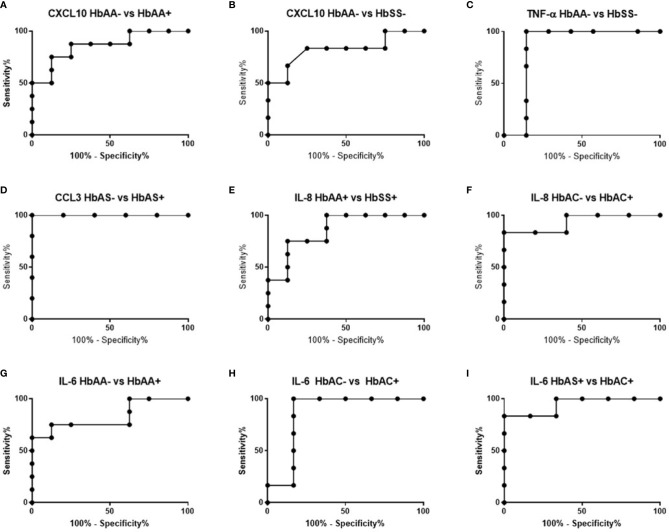
ROC analysis. **(A)** CXCL10; HbAA- vs HbAA+ (P = 0.02, AUC = 0.86). **(B)** CXCL10; HbAA- vs HbSS- (P = 0.05, AUC = 0.83). **(C)** TNF-α; HbAA vs HbSS- (P = 0.03, AUC = 0.86). **(D)** CCL3; HbAS- vs HbAS+ (P = 0.009, AUC = 1). **(E)** IL-8; HbAA+ vs HbSS+ (P = 0.02, AUC = 0.85). **(F)** IL-8; HbAC- vs HbAC+ (P = 0.02, AUC = 0.93). **(G)** IL-6; HbAA- vs HbAA+ (P = 0.03, AUC = 0.83). **(H)** IL-6; HbAC- vs HbAC+ (P = 0.04, AUC = 0.86). **(I)** IL-6; HbAS+ vs HbAC+ (P = 0.01, AUC = 0.94).

ROC curves and corresponding area under curve (AUC) assessments enable the prediction that CXCL10is independently discriminated between HbAA- and HbAA+ with an AUC = 0.86 and P = 0.02 (**Figure 2A**). CXCL10 and TNF-α independently discriminated between HbAA- and HbSS- with an AUC = 0.83 and P = 0.0.5 (**Figure 2B**) and AUC = 0.86 and P = 0.03 (**Figure 2C**), respectively. CCL3 independently discriminated between HbAS- and HbAS+ with an AUC = 0.1 and P = 0.0009 (**Figure 2D**). IL-8 independently discriminated between HbAA+ and HbSS+ with an AUC = 0.85 and P = 0.02 (**Figure 2E**) and HbAC- and HbAC+ with an AUC = 0.93 and P = 0.02 (**Figure 2F**). Lastly, IL-6 independently discriminated between HbAA- vs HbAA+ (P = 0.03, AUC = 0.83), HbAC- vs HbAC+ (P = 0.04, AUC = 0.86), and HbAS+ vs HbAC+ (P = 0.01, AUC = 0.94) (**Figures 2G–I**).

### Cytokine Concentrations Correlate With Hb, WBC, and RBC Counts in Individuals With Different Hemoglobin Genotypes Infected With or Without Malaria Parasites

**Figure 3:**
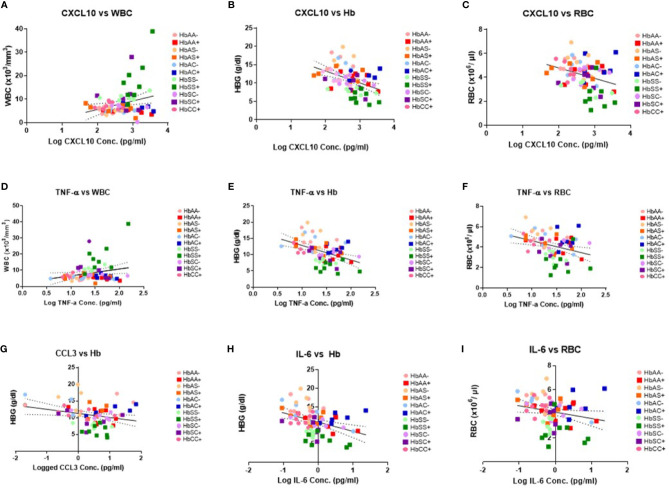
Correlations between cytokines, WBC and RBC counts, Hb levels, and Hb genotypes with (+) and without (-) malaria. A two-tailed Pearson Correlation with a 95% confidence interval and a linear regression was used for all comparisons. **(A)** Log transformed CXCL10 concentration vs WBC counts (R^2^ = 0.07, P = 0.02) and Y = 3.61X-1.55. **(B)** Log transformed CXCL10 concentration vs Hb levels (R^2^ = 0.19, P = 0.0001) and Y = 3.244X-19.83. **(C)** Log transformed CXCL10 concentration vs RBC counts (R^2^ = 0.11, P = 0.004) and Y = -0.868X+6.53. **(D)** Log transformed TNF-α concentration vs WBC counts (R^2^ = 0.06, P = 0.04) and Y = 4.239X+2.36. **(E)** Log transformed TNF-α concentration vs Hb levels (R^2^ = 0.02, P < 0.0001) and Y = -4.48+17.34. **(F)** Log transformed TNF-α concentration vs RBC counts (R^2^ = 0.11, P = 0.004) and Y = -1.16X +5.79. **(G)** Log transformed CCL2 concentration vs Hb levels (R^2^ = 0.07, P = 0.03) and Y = -1.22X+11.28. **(H)** Log transformed IL-6 concentration vs Hb levels (R^2^ = 0.15, P = 0.0007) and Y = -2.49X+10.88. **(I)** Log transformed IL-6 concentration vs RBC counts (R^2^ = 0.05, P = 0.047) and Y = -0.53X+4.14.

There were no significant correlations between the cytokines and PLT levels. However, correlations that were significant included: CXCL10 and WBC (R^2^ = 0.07, P = 0.02) (**Figure 3A**), CXCL10 and Hb (R^2^ = 0.19, P = 0.0001) (**Figure 3B**), CXCL10 and RBC (R^2^ = 0.11, P = 0.004) (**Figure 3C**), TNF-α and WBC (R^2^ = 0.06, P = 0.04) (**Figure 3D**), TNF-α and Hb (R^2^ = 0.02, P < 0.0001) (**Figure 3E**), TNF-α and RBC (R^2^ = 0.11, P = 0.004) (**Figure 3F**), CCL2 vs Hb (R^2^ = 0.07, P = 0.03 (**Figure 3G**), IL-6 vs Hb (R^2^ = 0.15, P = 0.0007) (**Figure 3H**), and IL-6 vs RBC (R^2^ = 0.05, P = 0.047) (**Figure 3I**). Significant correlations were identified for select individual genotypes with and without malaria between CXCL10, TNF-α and IL-6 with Hb, WBC, and RBC (**Supplement Tables 4–6**). HbSS+ for CXCL10 vs WBC (R^2^ = 0.63, P = 0.02) and TNF-α vs WBC (R^2^ = 0.58, P = 0.03) as well as for HbSC- for IL-6 vs WBC (R^2^ = 0.78, P = 0.02) were significantly correlated (**Supplement Table 4**). As well as, HbAC- for CCL2 vs Hb (R^2^ = 0.85, P = 0.03) and IL-6 vs Hb (R^2^ = 0.75, P = 0.03) while in HbAC+ for CCL3 (R^2^ = 0.72, P = 0.03) and in HbCC- for IL-8 (R^2^ = 0.76, P = 0.03) and CXCL10 (R^2^ = 0.52, P = 0.04) vs Hb (**Supplement Table 5**). Finally, n HbAC- for CCL2 vs RBC (R^2^ = 0.92, P = 0.01) as well as IL-6 vs RBC (R^2^ = 0.91, P = 0.003) and in HbCC- for CCL3 vs RBC (R^2^ = 0.71, P = 0.02) (**Supplement Table 6**).

### Ratios of Circulating Cytokine Concentrations Differ Among Individuals With Different Hemoglobin Genotypes Infected With or Without Malaria Parasites

**Figure 4:**
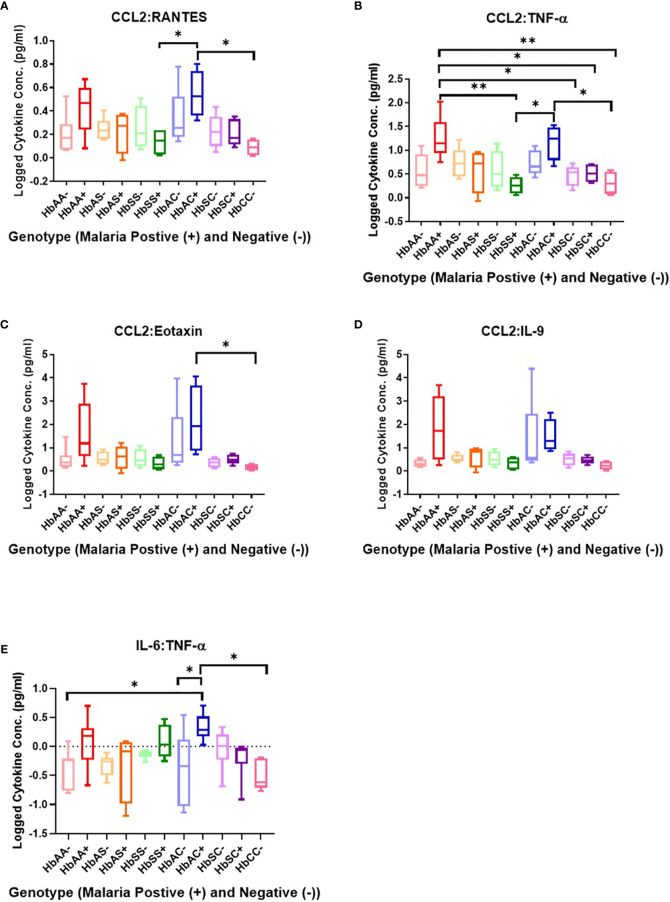
Assessment of cytokine concentration ratios in all Hb genotypes with (+) and without (-) malaria. A one-way ANOVA and Tukey’s multiple comparison test was used to assess between all the genotypes with (+) and without (-) malaria. **(A)** CCL2:RANTES HbSS+ (µ = 0.13) vs HbAC+ (μ = 0.54, P = 0.049) and HbAC+ (μ = 0.54) vs HbCC- (μ = 0.09, P = 0.02). **(B)** CCL2:TNF-α HbAA+ (μ = 1.24) vs HbSS+ (µ = 0.26, P = 0.003), HbAA+ (µ = 1.24) vs HbSC- (μ = 0.46, P = 0.02), HbAA+ (μ = 1.24) vs HbSC+ (μ = 0.51, P = 0.04), HbAA+ (μ = 1.24) vs HbCC- (μ = 0.31, P = 0.006), HbSS+ (μ = 0.26) vs HbAC+ (μ = 1.17, P = 0.01), R2 HbAC+ (μ = 1.17) vs HbCC- (μ = 0.31, P = 0.02). **(C)** CCL2:Eotaxin HbAC+ (μ = 2.16) vs HbCC- (μ = 0.17, P = 0.0498). **(D)** CCL2:IL-9 (= 0.35, P = 0.04) but there were no significant differences using the Tukey’s multiple comparison test. **(E)** IL-6:TNF-α HbAA- (μ = -0.4) vs HbAC+ (μ = 0.33, P = 0.03), HbAC- (μ = -0.38) vs HbAC+ (μ = 0.33, P = 0.049), and HbAC+ (μ = 0.33) vs HbCC- (μ = -0.49, P = 0.02). *: 0.049-0.01, **: 0.009-0.001.

**Figure 5:**
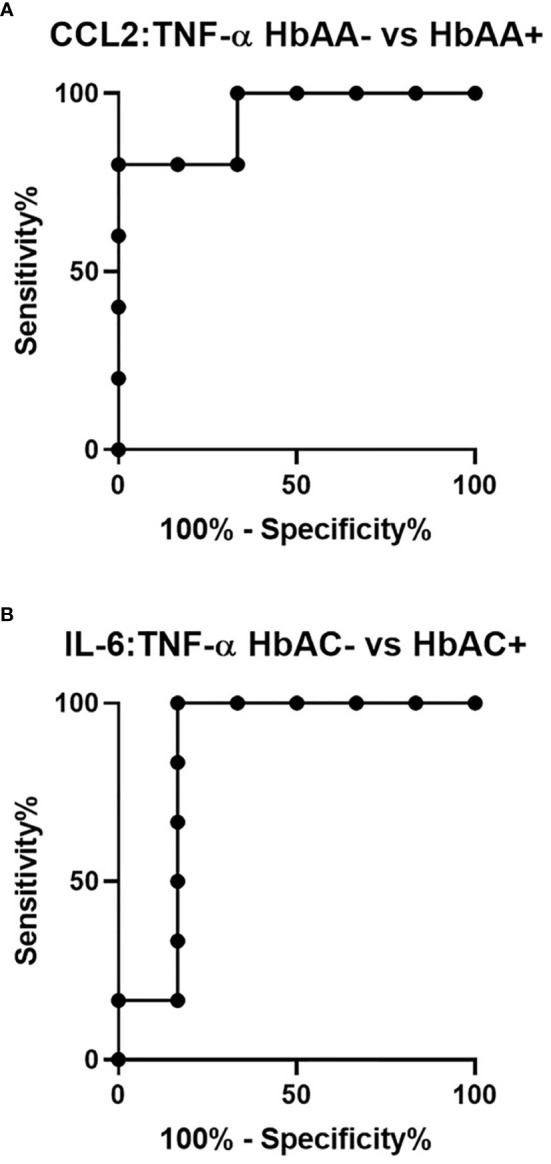
ROC analysis for CCL2:TNF-α and IL-6:TNF-α. **(A)** CCL2:TNF-α independently discriminate between HbAA- vs HbAA+ with an AUC = 0.93 and P = 0.02. **(B)** IL-6:TNF-α independently discriminate between HbAC- vs HbAC+ with an AUC = 0.86 and P = 0.04.

Ratios were assessed in individuals and compared among hemoglobin genotypes infected with or without malaria (**Figure 4**). While multiple cytokine ratios had significant difference between groups the ratios of CCL2 to TNF-α was significant between HbAA+ (µ = 1.24) vs HbAA- (µ = 0.55) (P = 0.02) (**Figure 4B**) and for the IL-6 to TNF-α ratio, there was a significant difference for HbAC- (µ = -0.38) vs HbAC+(µ = 0.33) (**Figure 4E**) had significant ROC curves. The ratio of CCL2 to TNF-α independently discriminated between HbAA- and HbAA+ with an AUC = 0.93 and P = 0.02 (**Figure 5A**). The ratio of IL-6 to TNF-α independently discriminated between HbAC- and HbAC+ with an AUC = 0.86 and P = 0.04 (**Figure 5B**).

### Principal Component Analysis of 6 Cytokines in Relation to WBC, RBC, Hb, and PLT for All Groups With and Without Malaria

**Figure 6:**
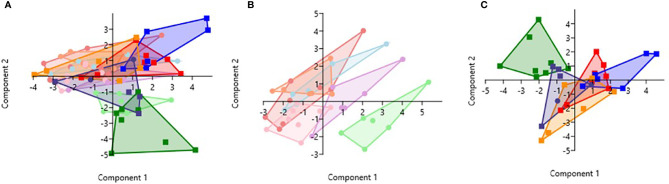
Principal Component Analysis for cytokines (CXCL10, CCL2, TNF-α, CCL3, IL-8, IL-6) and CBC (WBC, RBC, Hb, and PLT). Principal Component Analysis was applied to cytokines (CXCL10, CCL2, TNF-α, CCL3, IL-8, IL-6) and CBC (WBC, HCT, Hb, and PLT) variables in Past 3.x (23). A correlation matrix was used since variables were measured in different units as well as between-group analysis. Missing data was replaced with column average using mean value imputation. Red is for HbAA-/+, orange is for HbAS-/+, blue is for HbAC-/+, purple is for HbSC-/+, green is for HbSS-/+, and pink is for HbCC-. Pastel color represents malaria negative and dark colors represent malaria positive groups. **(A)** Illustrates all genotypes with and without malaria. The PC1 and PC2 eigenvalue and percent variance are 4.9, 49% and 4.1, 40.6%, respectively. **(B)** Illustrates malaria negative genotypes only. PC1 and PC2 eigenvalue (5.82, 2.5) and percent variance (58.2, 25.3%), respectively. **(C)** Illustrates malaria positive genotypes only. PC1 eigenvalue and percent variance are 5.94 and 59.5%, respectively. PC2 eigenvalue and percent variance are 3.8 and 38.2%, respectively.

PCA analysis revealed that HbSS- and HbSS+ groups distinctively cluster away from other hemoglobin genotypes (**Figure 6**). The scoring plot shows HbSS-/+ can be differentiated from other genotypes based on the first principal component (PC1), while other Hb groups cluster in the sample regions. **Figure 6A** contains all genotypes with and without malaria. **Figure 6A** has an eigenvalue (variance in the data on that axis), and percent variance for PC1 (4.9, 49%) and PC2 (4.1, 40.6%), respectively. HbSS-/+ cluster together and slightly away from other genotypes with and without malaria. When only uninfected groups were analyzed, eigenvalue and percent variance for PC1 (5.82, 58.2%) and PC2 (2.5, 25.3%), and the HbSS- clusters are close together and distant from other groups. Finally, HbSS+ clustered away from other groups (**Figure 6C**) among malaria positive genotypes showing an eigenvalue and percent variance for PC1 (5.94, 59.5%) and PC2 (3.8, 38.2%).

## Discussion

The study identified unique and remarkable correlations between hemoglobin genotype, malaria status, and circulating inflammatory marker levels. Inflammatory chemokines CXCL10, CCL2, and CCL3 as well as cytokines, TNF-α, IL-8, and IL-6 were differentially expressed in individuals with different hemoglobin genotypes that were infected or uninfected with malaria parasites (**Figures 1**, **Supplement Figure 1**). Also, CXCL10, CCL3, TNF-α, and IL-6 levels were tightly associated with WBC, RBC counts, and Hb levels (**Figure 3**, **Supplement Tables 4–6**). Other studies have indicated that some of the inflammatory factors identified here could be used as predictive markers for SCD and malaria severity (24, 25). Thus, our results suggest that chemokines CXCL10 and CCL2 and cytokines, TNF-α, IL-8, and IL-6 may contribute to the overall pro-inflammatory response and could be utilized for predicting the severity of *Plasmodium* infection or a hemoglobinopathy (**Figure 2**). Furthermore, it was determined that the ratio of CCL2 concentration to TNF-α and IL-6 to TNF-α could also potentially predict the severity of *Plasmodium* infection (**Figure 4**).

Patient demographics revealed multiple differences between hemoglobin genotype and infection with malaria (**Supplement Table 1**). The most remarkable differences in CBC levels were observed in HbSS- and HbSS+ groups (**Supplement Table 1**). These results may be because of SCD-associated inflammation causing increased WBC and PLT counts as well as low levels of RBC counts and Hb (10, 26, 27). SCD individuals have higher WBC and PLT levels driven by chronic inflammation associated with SCD (10). In HbSS patients, RBCs have a short life span due to the sickling of the RBC, and destruction of RBC by hemolysis leads to low RBC counts and Hb levels and anemia (10, 11). The differences in the CBC level identified between malaria and non-malaria groups could be associated with factors mediating malaria pathogenesis (**Supplement Table 1**). As observed in SCD, RBCs are destroyed at a faster rate than in non-SCD individuals due to the parasite replication cycle and may explain the lower levels of RBC and Hb seen in malaria positive groups compared to malaria negative groups (1).

In the non-malaria groups, assessment of cytokine levels in uninfected Hb genotypes confirmed multiple roles of CXCL10 as a chemoattractant for monocytes and T cells and promoter of T cell adhesion to endothelial cells (22). CXCL10 functions as a T and natural killer (NK) cell trafficker (28). CXCL10 is elevated in HbSS- compared to HbAA- and independently discriminated between HbAA- and HbSS-, and was also significantly elevated in HbSC- compared to HbAA- (**Figure 4**, **Supplement Figure 1**). Previous studies have reported an important role of CXCL10 in SCD pathogenesis (29–31). A study assessing cytokine levels in SCA patients with and without albuminuria reported higher levels of CXCL10, CCL2, and IL-8 (also known as C-X-C motif ligand 8 CXCL8) in urine samples of children with albuminuria (31). CXCL10 is associated with severity in SCD. Therefore, alterations in CXCL10 levels in individuals with different Hb genotypes provides insight into the role of CXCL10 in SCD pathogenesis. When assessing malaria positive groups, the results indicated that CXCL10 levels were significantly elevated in HbAA+ vs HbAA- and for HbAS+ vs HbAS-. CXCL10 could be independently discriminated between HbAA+ vs HbAA- (**Figures 1**, **2**, **Supplement Figure 2**). We also determined that circulating CXCL10 concentration correlated with RBC, WBC counts, and Hb (**Figure 3**) in concert with the tight association between CXCL10 and malaria pathogenesis (22). Increased expression of CXCL10 above basal levels has been linked to fatal cerebral malaria (28, 32, 33). The differential expression of CXCL10 in different Hb genotypes suggests a role of Hb genotype in RBC and WBC counts as well as Hb levels and CXCL10 expression. This could suggest potential crosstalk between inflammatory pathways and Hb genotype that may regulate malaria outcomes. There may be hitherto unreported crosstalk between signaling pathways of inflammatory factors, RBC and WBC counts, and Hb genotypes that may regulate malaria outcome.

TNF-α activates endothelial cells and neutrophils (34). In the non-malaria conditions, TNF-α was significantly elevated in HbSS- compared to HbAA- (**Supplement Figure 1**). In the literature, TNF-α increases the risk of stroke in SCA patients, and SCA patients with higher levels of TNF-α had more frequent leg ulcers, acute chest syndrome, femoral necrosis, and recurrent infection (34). Since TNF-α independently discriminated between HbAA- vs HbSS- (**Figure 4**) it seems that the differential expression of TNF-α could be a result of differential activation of endothelial cells and neutrophils driven by hemoglobin status and that TNF-α may be a potential biomarker of HbSS. Understanding the impact of different cytokine profiles on each Hb genotype allows for a better understanding of the correlation between Hb genotypes and protection against malaria. However, when assessing TNF-α and Hb genotypes when compared with and without malaria there was no tight association, but there was an increasing trend in TNF-α levels in malaria positive groups (**Figure 1**). Higher plasmaconcentrations of TNF-α were reported in severe malaria cases compared to uncomplicated malaria in Sri Lanka (35). One study in mice reported a reduction in CXCL10, TNF-α, and CCL2 levels when treated with artesunate and recombinant human erythropoietin and a resulting increase in survival rates and improved blood-brain barrier integrity (36). Therefore, CXCL10 and TNF-α may be good candidates for predicting the severity of HbSS based on inflammation response compared to HbAA individuals.

CCL2 functions in monocyte trafficking and CCL3 functions in macrophage and NK cell migration as well as T-cell and dendritic cell (DC) interactions (28). Comparative analysis of CCL2 levels in different Hb genotypes revealed multiple significant difference between HbAC+ and other groups (**Figure 1**). These differences in CCL2 levels may be a result of the remarkable increase in HbAC+ individuals. This may indicate in HbAC individuals CCL2 plays a role in their protective mechanism against malaria. CCL3 was significantly elevated in HbAS+ compared to HbAS-and independently distinguished HbAS- and HbAS+ (**Figure 3**, **Supplement Figure 1**). CCL3 was also significantly elevated in HbAC+ compared to HbAC- (**Supplement Figure 1**). Indicating that the protection against malaria associated with this genotype may be mediated by CCL3.

IL-8 mediates neutrophil trafficking and IL-6 has a “pleiotropic effect on inflammation, immune response, and hematopoiesis” as well as has been associated with acute phase response in malaria (28, 37, 38). There was no significant difference reported between Hb genotypes without malaria for IL-8. It was determined that IL-8 was significantly elevated in HbAC+ compared to HbAC and independently discriminated between HbAC- and HbAC+ (**Figure 3**, **Supplement Figure 1**). Previous studies have reported increased levels of CCL3, as well as CCL2 and IL-8, have been reported in pregnant women with malaria (39, 40). IL-6 has been reported to be elevated in SCD (41). There was no significant increase in IL-6 in HbSS- and HbSC- compared to HbAA-. The lack of a clear difference could be a result of the variation seen in the levels of IL-6 for HbAA- individuals whereas IL-6 levels in HbSS- and HbSC- individuals are more tightly grouped. IL-6 was significantly elevated and independently discriminated between HbAA+ vs HbAA-, HbAC+ vs HbAC-, and HbAC+ vs HbAS+ (**Figures 1****–****3**, **Supplement Table 3**). IL-6 was reported to be elevated in children with malaria and SCD (37, 41). Based on our data CXCL10, TNF-α, CCL3, IL-8, and IL-6 may be investigated as potential markers to assess malaria severity and protection.

Assessment of cytokine concentrations across all groups with or without malaria indicated differential expression of inflammatory cytokines (**Figure 1**, **Supplement Figure 1**). For example, IL-6 concentration was correlated with WBC count for HbSC-. These correlations may be derived from the association of IL-6 with hematopoiesis (38). Principal component analysis (PCA; **Figure 6**) assessing CBC levels as well as cytokine and chemokine levels indicated that the HbSS-/+ genotypes cluster together and away from other groups when all factors associated with malaria or not being considered. The HbCC- group clustered away from other groups indicating that these factors mediate effects observed in different Hb genotypes. Finally, the only two cytokine ratios that could be used to assess disease severity were those of CCL2/TNF-α in HbAA- vs HbAA+ and IL-6/TNF-α in HbAC- vs HbAC+ (**Figure 6**). Therefore, the mediating mechanisms need further investigation to enable the development of severity markers for malaria and SCD.

In conclusion, some cytokines are differentially expressed among individuals with different hemoglobin genotypes infected with or without malaria parasites. Circulating CXCL10, CCL3, IL-8, TNF-α, and IL-6 could be used as potential biomarkers for malaria severity among individuals with hemoglobinopathies. CXCL10 and TNF-α could be used to differentiate HbAA from HbSS. It was also determined that malaria outcomes could be altered based on the crosstalk between inflammatory cytokines (CXCL10, CCL3, IL-8, TNF-α, and IL-6) and Hb genotypes. Assessing cytokine levels among individuals with different hemoglobinopathies increases our understanding of the pathogenesis of severe malaria in different hemoglobinopathies. Assessing cytokine levels will also allow us to determine potential biomarkers of disease severity in individuals with malaria and hemoglobinopathies. However, the small sample size limited our ability to assess the specificity and the sensitivity of the proposed markers. For that reason, more studies are required on a larger sample size to determine the validity of the markers identified for assessing malaria and sickle cell disease severity. The use of such markers could facilitate a deeper understanding of both disorders and facilitate rapid treatment implementation in malaria patients to decrease disease severity, comorbidities, and recovery time.

## Data Availability Statement

The raw data supporting the conclusions of this article will be made available by the authors, without undue reservation.

## Ethics Statement

The studies involving human participants were reviewed and approved by Morehouse School of Medicine, College of Health Sciences, University of Ghana, and the Noguchi Memorial Institute for Medical Research at University of Ghana. Written informed consent to participate in this study was provided by the participants’ legal guardian/next of kin.

## Author Contributions

AD, JS, and KH designed the study. AD, MW, YD-A, and FB provided support in subject enrollment and sample collection. KH and AD conducted the experiments. KH, AD, and JS analyzed and interpreted the data. KH and AD wrote the paper. FB, MW, YD-A, AA, JH, JS, and AD edited and approved the final manuscript. All authors contributed to the article and approved the submitted version.

## Funding

The study was supported by National Institutes of Health, grant numbers NIH/FIC 1K01TW010282 (Driss, PI), NIH/FIC UJMT Fogarty Global Health Fellows Program #5R25TW009340 (Chi,PI), NIH/NIMHD Research Centers in Minority Institutions (RCMI) 5U54MD007602 (Bond, PI; Driss pilot PI), National Center for Advancing Translational Sciences of the National Institutes of Health TL1TR002382 (Harp, TL1 Trainee), NIH/NINDS 1R01NS091616 (Stiles, PI) and National Institute on Minority Health and Health Disparities G12MD007602 (Bond, PI), and NIH 2U54MD007602. The Elsa U. Pardee Foundation (Hood, PI), University of Louisville Department of Pharmacology and Toxicology Faculty Start-up funds (Hood, PI) and UofL COBRE NIGMS NIH P20GM113226 (McClain PI, Hood pilot PI) are also recognized for supporting J. L. Hood.

## Conflict of Interest

The authors declare that the research was conducted in the absence of any commercial or financial relationships that could be construed as a potential conflict of interest.

## Supplementary Material

Supplementary Figure 1 Comparisons cytokines for genotypes with and without malaria using t-tests. Graphs are box and whisker plots showing minimum and maximum. **(A)**, Unpaired two-tailed t-test for CXCL10 was used to compare HbAA- vs HbSS- (P = 0.04) and HbAA- vs HbSC- (P = 0.02). **(B)**, Unpaired two-tailed t-test for CXCL10 was run in HbAA- vs HbAA+ (P = 0.01), HbAA- vs HbSS+ (P = 0.0001), and HbAC- vs HbAC+ (P = 0.03). **(C)**, A two-tailed Mann-Whitney test was used to compare CCL2 in HbAA- vs HbSS+ (P = 0.04). **(D)**, A two-tailed Mann-Whitney test was used to compare TNF-α in HbAA- vs HbSS- (P = 0.03). **(E)**, An unpaired two-tailed t-test was used to compare CCL3 in HbAC- vs HbAC+ (P = 0.03) and a Mann-Whitney test used to compare HbAS+ vs HbAS+ (P = 0.008). **(F)**, An unpaired two-tailed t-test was used to compare IL-8 in HbAC- vs HbAC+ (P = 0.01) as well as HbAA+ vs HbSS+ (P = 0.007). **(G)**, An unpaired two-tailed t-test was used to compare IL-6 in HbAA- vs HbAA+ (P = 0.01) as well as HbAC- vs HbAC+ (P = 0.03).
