# Supplementary material for: Hemoglobin Genotypes Modulate Inflammatory Response to Plasmodium Infection
Source: Front Immunol. 2020 Dec 23;11:593546. doi: 10.3389/fimmu.2020.593546 (PMC7786007; doi:10.3389/fimmu.2020.593546)
Supplement: Supplementary Figure 1 — Comparisons cytokines for genotypes with and without malaria using t-tests. Graphs are box and whisker plots showing minimum and maximum. (A), Unpaired two-tailed t-test for CXCL10 was used to compare HbAA- vs HbSS- (P = 0.04) and HbAA- vs HbSC- (P = 0.02). (B), Unpaired two-tailed t-test for CXCL10 was run in HbAA- vs HbAA+ (P = 0.01), HbAA- vs HbSS+ (P = 0.0001), and HbAC- vs HbAC+ (P = 0.03). (C), A two-tailed Mann-Whitney test was used to compare CCL2 in HbAA- vs HbSS+ (P = 0.04). (D), A two-tailed Mann-Whitney test was used to compare TNF-α in HbAA- vs HbSS- (P = 0.03). (E), An unpaired two-tailed t-test was used to compare CCL3 in HbAC- vs HbAC+ (P = 0.03) and a Mann-Whitney test used to compare HbAS+ vs HbAS+ (P = 0.008). (F), An unpaired two-tailed t-test was used to compare IL-8 in HbAC- vs HbAC+ (P = 0.01) as well as HbAA+ vs HbSS+ (P = 0.007). (G), An unpaired two-tailed t-test was used to compare IL-6 in HbAA- vs HbAA+ (P = 0.01) as well as HbAC- vs HbAC+ (P = 0.03). [file Presentation_1.pptx]

## Slide 1
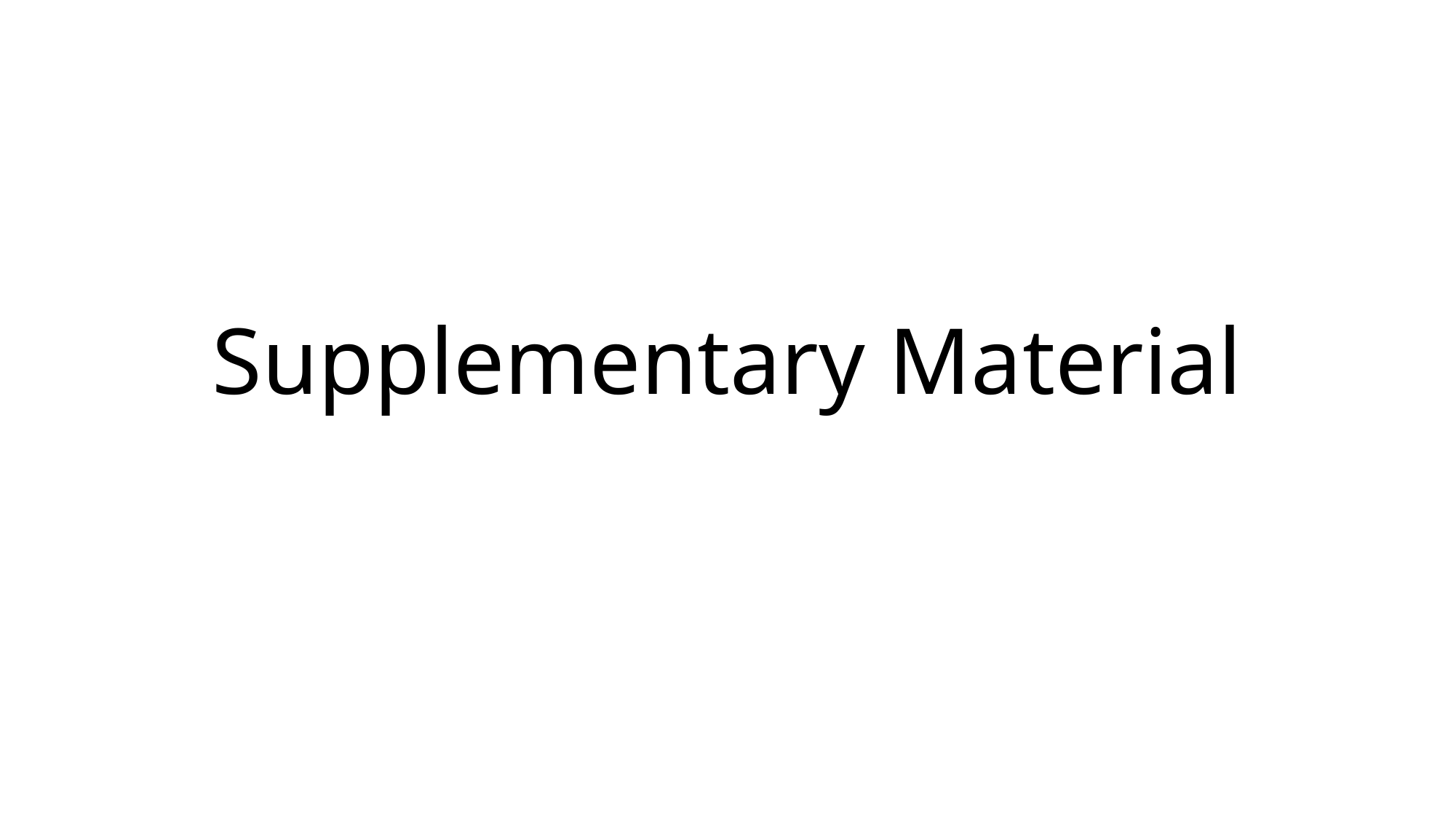

# Supplementary Material

## Slide 2
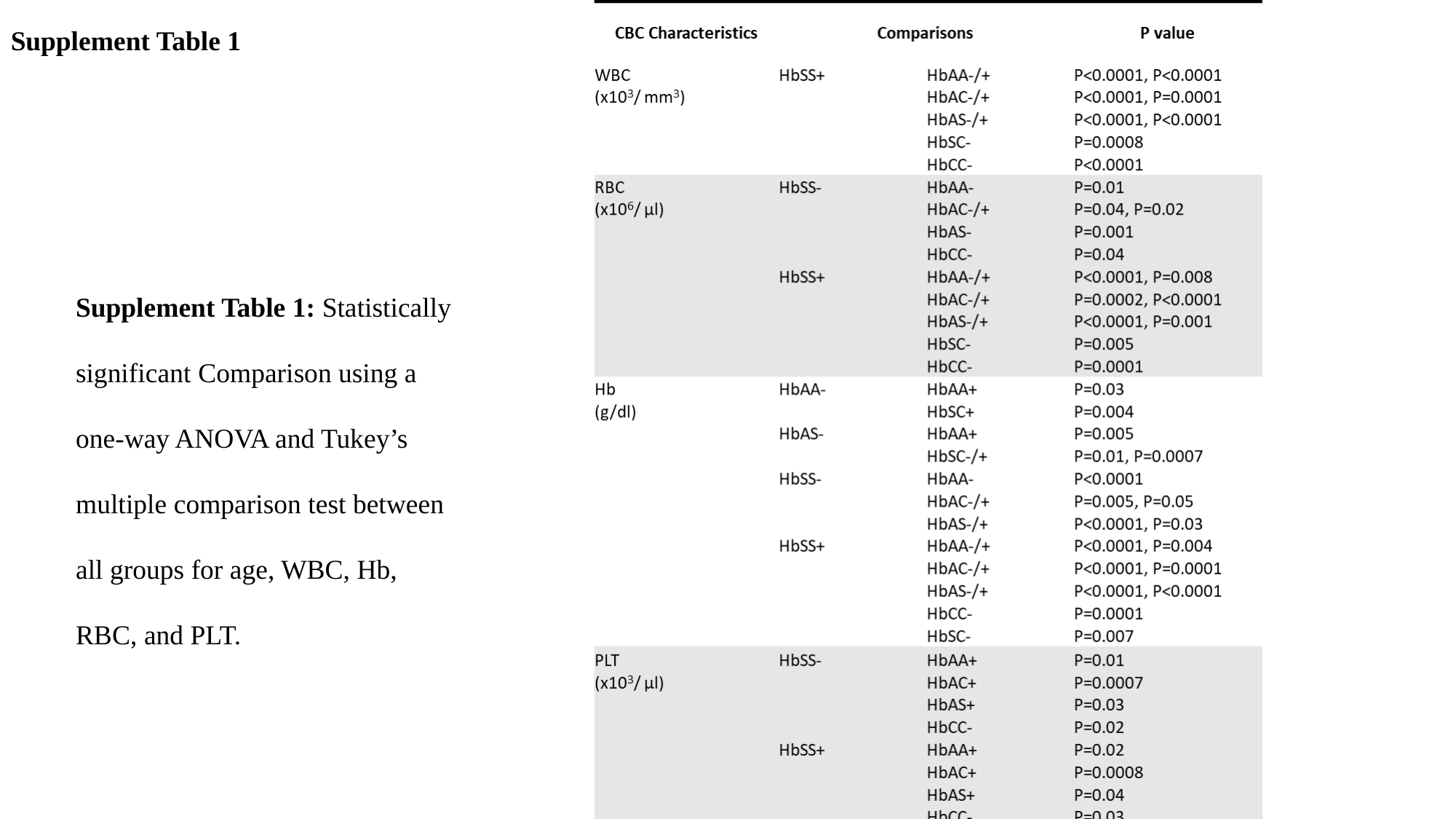

Supplement Table 1
Supplement Table 1: Statistically significant Comparison using a one-way ANOVA and Tukey’s multiple comparison test between all groups for age, WBC, Hb, RBC, and PLT.

## Slide 3
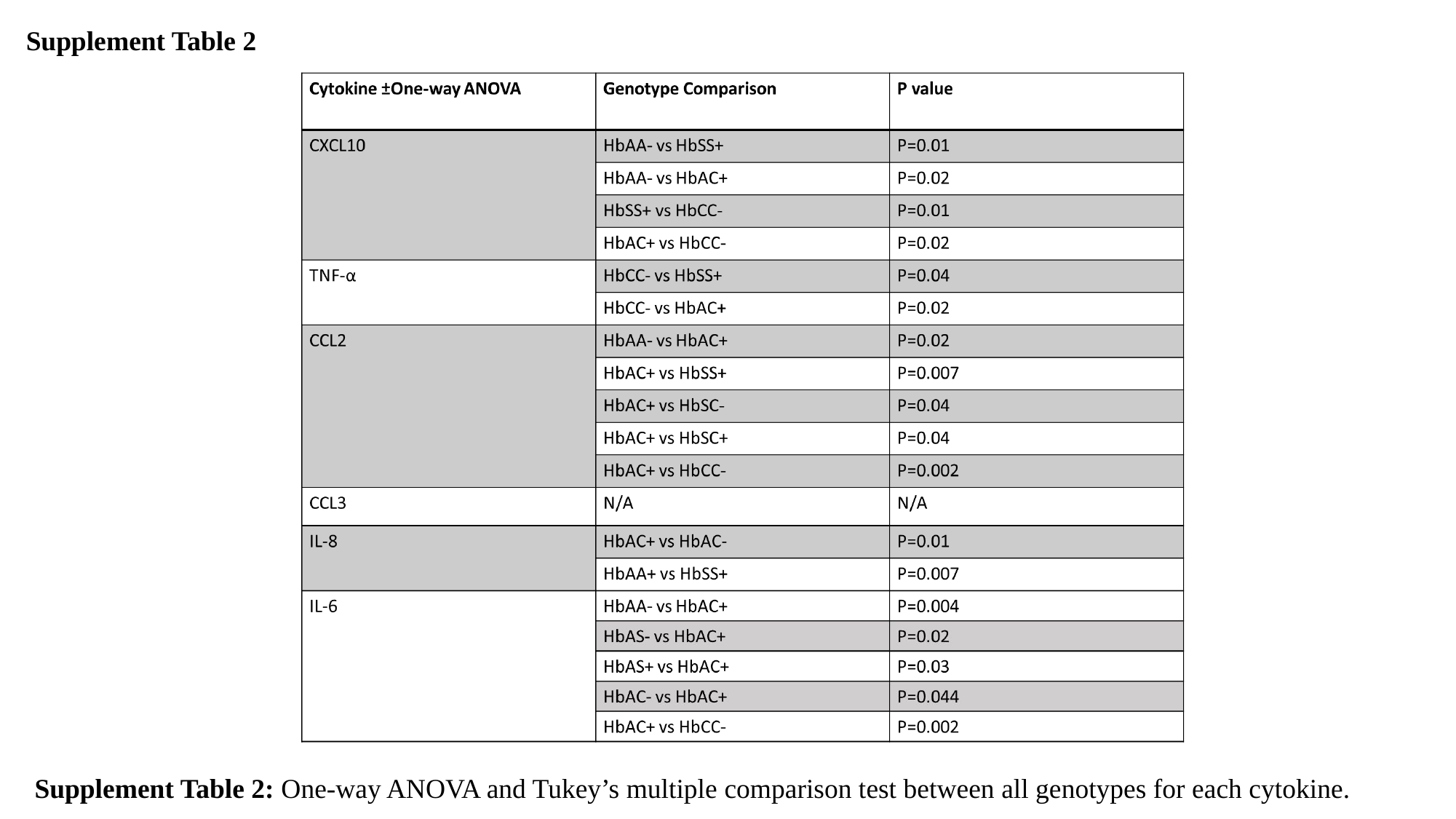

Supplement Table 2
Supplement Table 2: One-way ANOVA and Tukey’s multiple comparison test between all genotypes for each cytokine.

## Slide 4
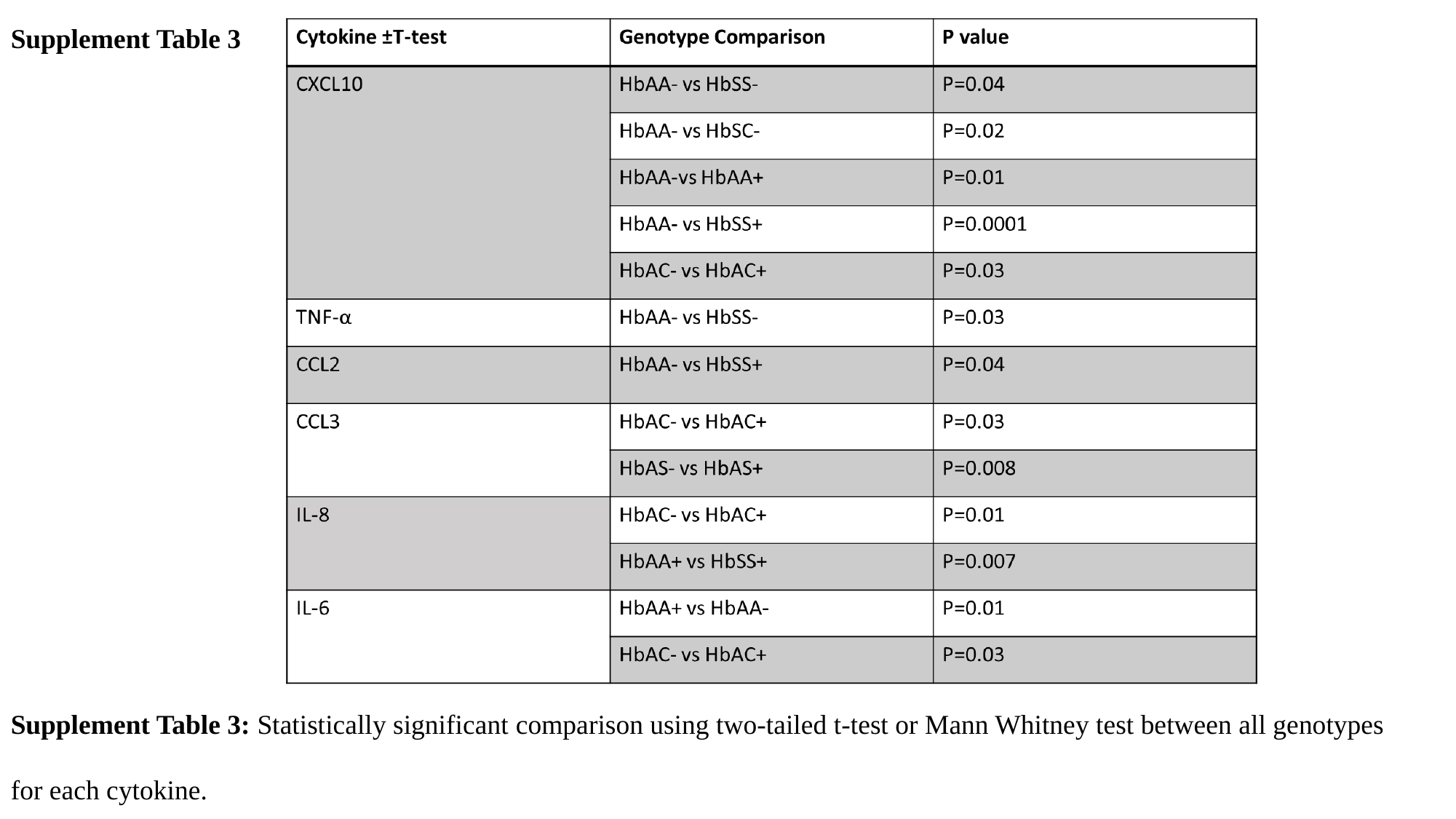

Supplement Table 3
Supplement Table 3: Statistically significant comparison using two-tailed t-test or Mann Whitney test between all genotypes for each cytokine.

## Slide 5
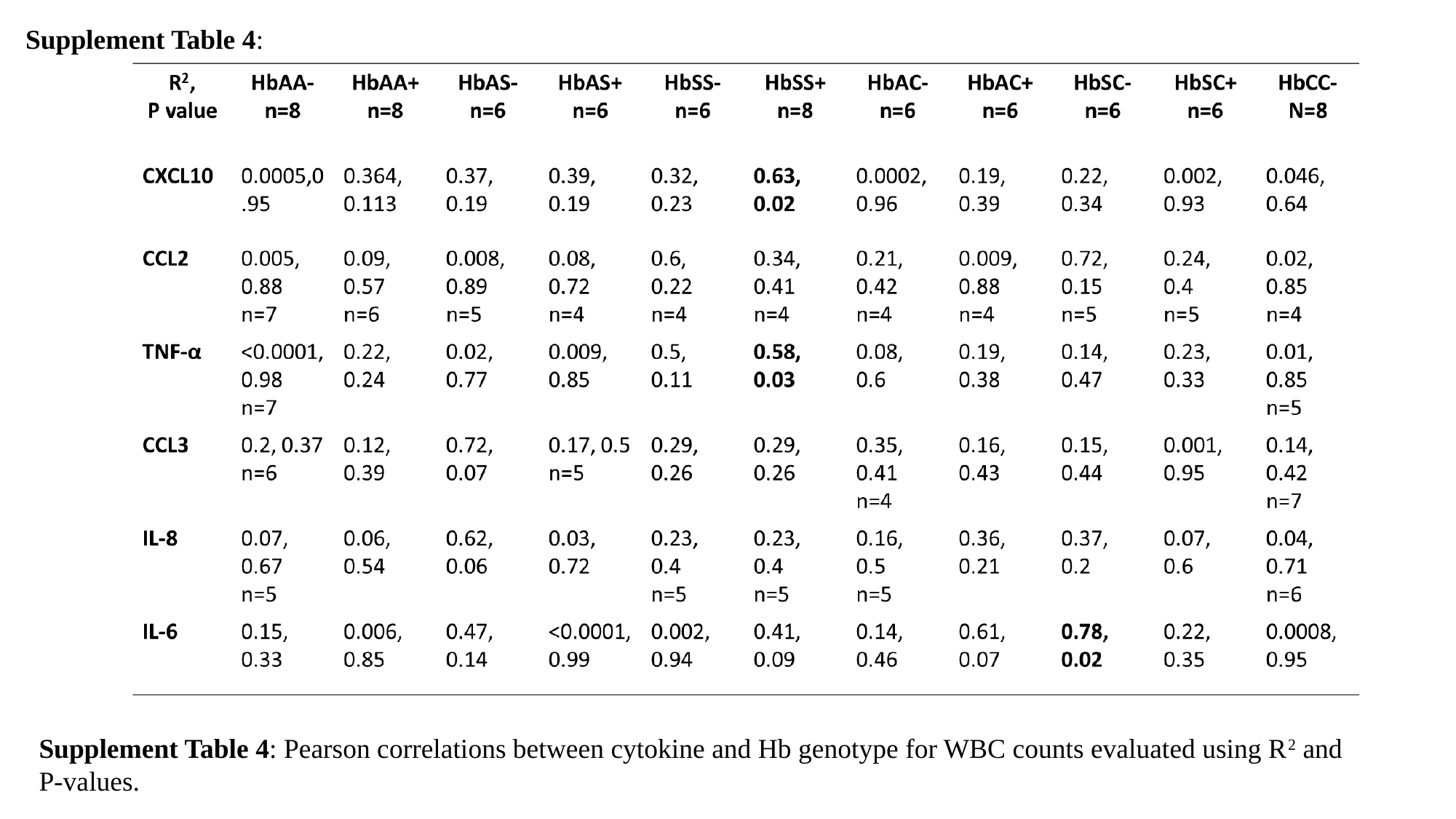

Supplement Table 4:
Supplement Table 4: Pearson correlations between cytokine and Hb genotype for WBC counts evaluated using R2 and P-values.

## Slide 6
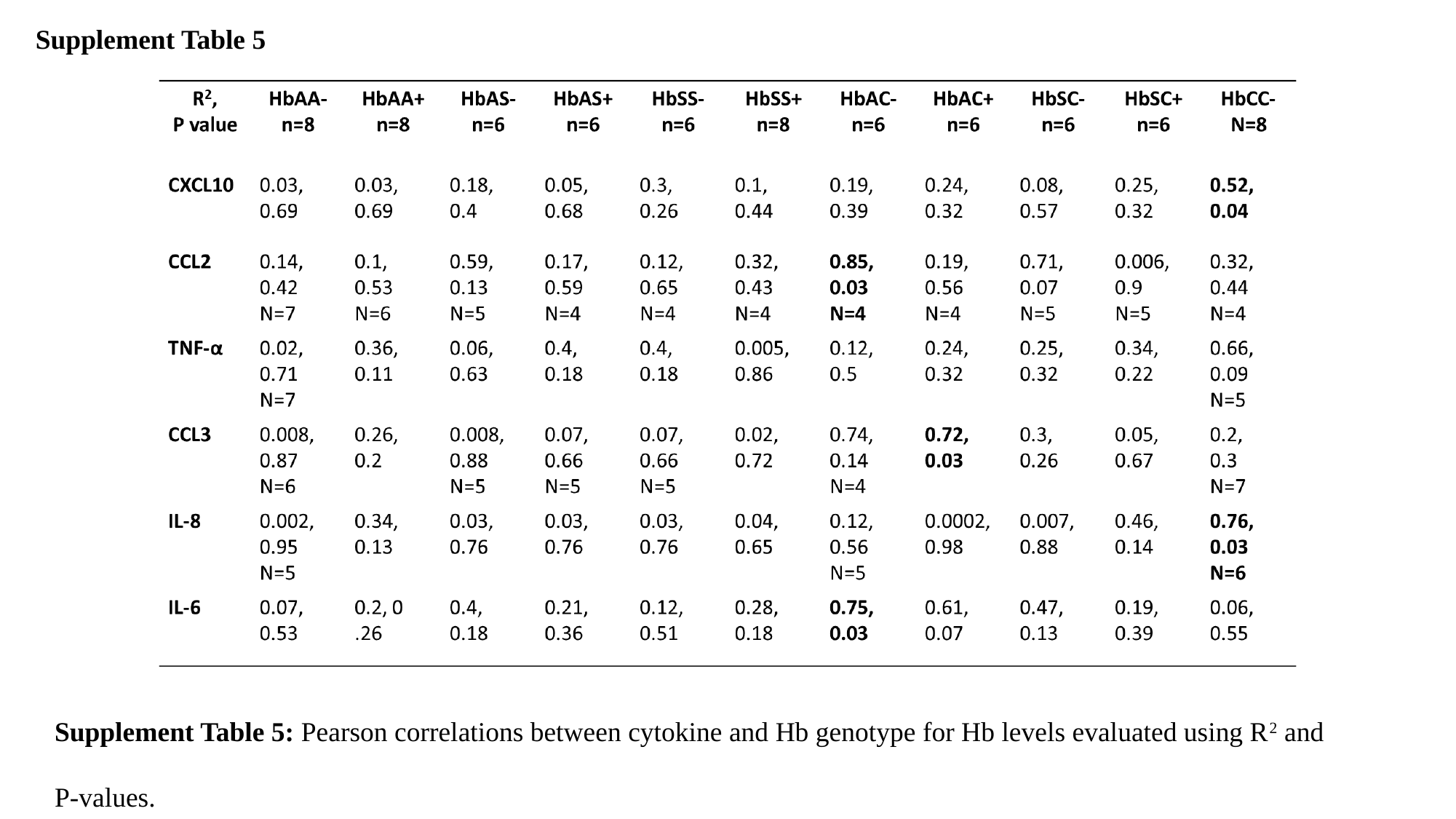

Supplement Table 5
Supplement Table 5: Pearson correlations between cytokine and Hb genotype for Hb levels evaluated using R2 and P-values.

## Slide 7
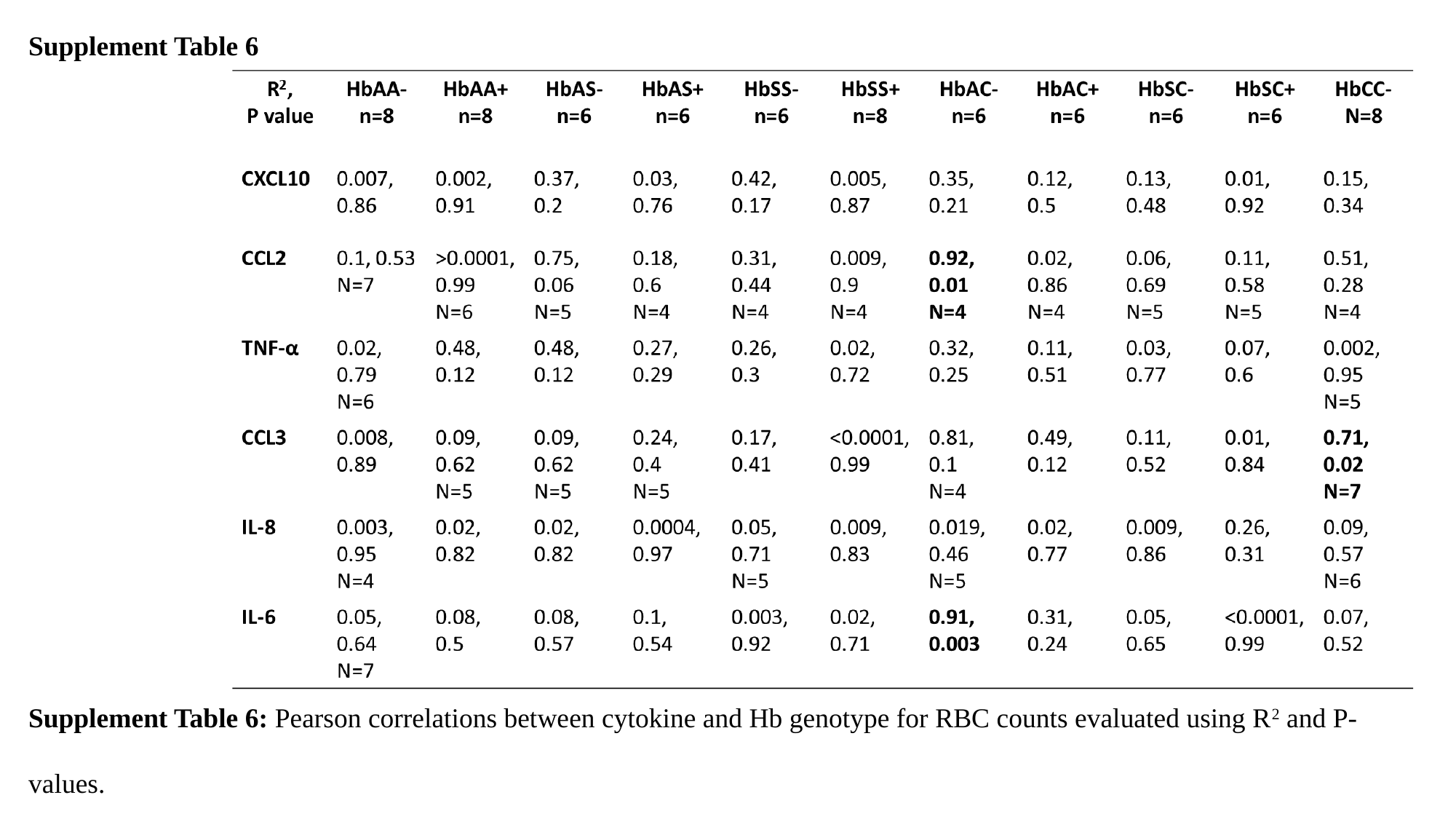

Supplement Table 6
Supplement Table 6: Pearson correlations between cytokine and Hb genotype for RBC counts evaluated using R2 and P-values.

## Slide 8
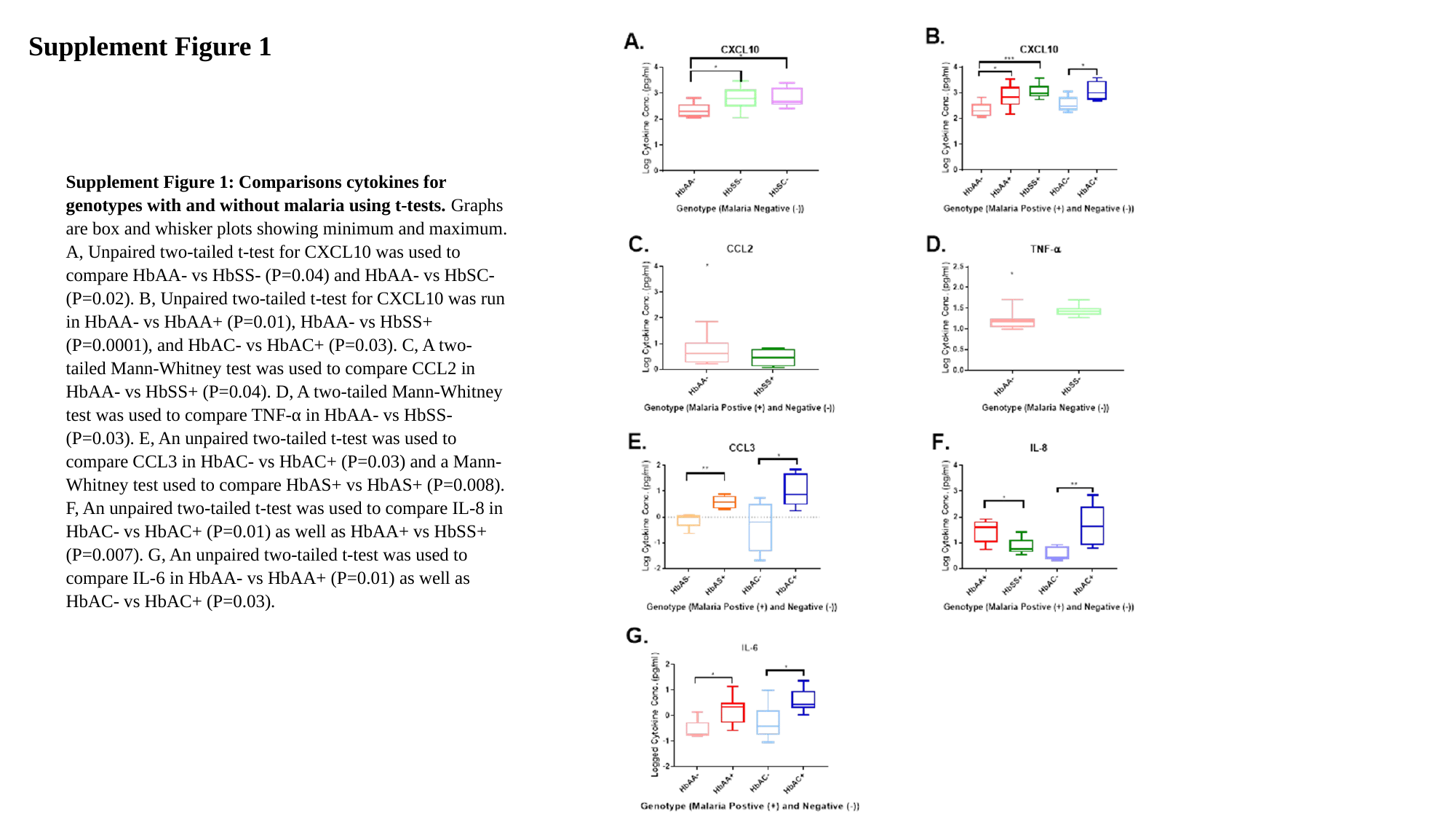

Supplement Figure 1
Supplement Figure 1: Comparisons cytokines for genotypes with and without malaria using t-tests. Graphs are box and whisker plots showing minimum and maximum. A, Unpaired two-tailed t-test for CXCL10 was used to compare HbAA- vs HbSS- (P=0.04) and HbAA- vs HbSC- (P=0.02). B, Unpaired two-tailed t-test for CXCL10 was run in HbAA- vs HbAA+ (P=0.01), HbAA- vs HbSS+ (P=0.0001), and HbAC- vs HbAC+ (P=0.03). C, A two-tailed Mann-Whitney test was used to compare CCL2 in HbAA- vs HbSS+ (P=0.04). D, A two-tailed Mann-Whitney test was used to compare TNF-α in HbAA- vs HbSS- (P=0.03). E, An unpaired two-tailed t-test was used to compare CCL3 in HbAC- vs HbAC+ (P=0.03) and a Mann-Whitney test used to compare HbAS+ vs HbAS+ (P=0.008). F, An unpaired two-tailed t-test was used to compare IL-8 in HbAC- vs HbAC+ (P=0.01) as well as HbAA+ vs HbSS+ (P=0.007). G, An unpaired two-tailed t-test was used to compare IL-6 in HbAA- vs HbAA+ (P=0.01) as well as HbAC- vs HbAC+ (P=0.03).
